# Holoprosencephaly in Patau Syndrome

**DOI:** 10.1590/1984-0462/2023/41/2022027

**Published:** 2023-03-13

**Authors:** Amanda de Souza Schlosser, Giovani José Coury Costa, Henrique Salmazo da Silva, Juan Luca Menezes de Mello, Lucy de Oliveira Gomes, Marina Michalski Oliveira Onoyama, Tatiana Martins Coury Costa

**Affiliations:** a Universidade Católica de Brasília, Brasília, DF, Brazil.; b Centro Universitário do Planalto Central Apparecido dos Santos, Brasília, DF, Brazil.; c Tatiana Medicina e Imagem, Brasília, DF, Brazil.

**Keywords:** Trisomy 13 syndrome, Patau syndrome, Chromosome 13 duplication, Holoprosencephaly, Semilobar holoprosencephaly, Congenital abnormalities, Síndrome da trissomia do cromossomo 13, Síndrome de Patau, Duplicação do cromossomo 13, Holoprosencefalia, Holoprosencefalia semilobar, Anormalidades congênitas

## Abstract

**Objective:**

To evaluate radiological (gestational and perinatal) and neonatal signs of patients with Patau syndrome and semilobar holoprosencephaly, as well as to report the association of both pathologies.

**Case description:**

This case report is about a female infant, born at term with trisomy of the chromosome 13 and semilobar holoprosencephaly, with thalamic fusion and a single cerebral ventricle, in addition to several other changes that worsened the patient's prognosis.

**Comments:**

Chromosome 13 trisomy is a genetic alteration that leads to the symptoms that determines Patau syndrome. In this syndrome, cardiovascular, urogenital, central nervous system, facial structure and intellectual impairment are common, in addition to problems in limb formation, such as decreased humerus and femur length, polydactyly, hypotelorism and low ear implantation. It is estimated, however, that holoprosencephaly is present in only 24 to 45% of the patients with trisomy 13.

## RESUMO

**Objetivo:**

Avaliar sinais radiológicos (gestacionais e perinatais) e neonatais de paciente com síndrome de Patau e holoprosencefalia semilobar, assim como relatar a associação de ambas as patologias.

**Descrição do caso:**

Trata-se de um relato de recém-nascido do sexo feminino a termo, que apresentou trissomia do cromossomo 13 e holoprosencefalia semilobar, com fusão talâmica e ventrículo cerebral único, além de várias outras alterações que pioraram o prognóstico da paciente.

**Comentários:**

A trissomia do cromossomo 13 é um defeito genético que caracteriza um conjunto de sintomas que compõem a Síndrome de Patau. Nesta síndrome, é comum o acometimento cardiovascular, urogenital, do sistema nervoso central, da estrutura facial e da capacidade intelectual, além de falhas na formação dos membros, como diminuição no comprimento do úmero, fêmur, polidactilia, hipotelorismo e baixa implantação das orelhas. Estima-se, no entanto, que a holoprosencefalia apresente-se nesse grupo de malformações congênitas apenas em 24 a 45% dos casos.

## INTRODUCTION

Patau syndrome is caused by the trisomy of chromosome 13. This syndrome can be diagnosed at birth or during prenatal care, and the main findings are microphthalmia, polydactyly and cleft palate.^1^ This pathology is estimated at approximately 1:20,000 live births, with a life expectancy of 7 to 10 days. Most cases have early lethality as a result of abortions prior to diagnosis. Approximately 91% of neonates do not exceed the first year of life, while those who pass this mark face serious problems in cognitive development and growth, seizures and episodes of apnea. In addition to these alterations, common signs of Patau syndrome are heart defects, kidney disorders, microcephaly and defects in the genesis of auditory structures, such as deafness and low implantation of the ears.^1^

Holoprosencephaly (HPE), in turn, is a defect in the cleavage of the forebrain, occurring between the 18th and 28th week of gestation, caused by failures in the action of signaling molecules such as Hedgehog (Hh) protein precursor, fibroblast growth factor (FGF), transforming growth factor-beta (TGFß) and WnT signaling pathway that regulate the transcription of genes responsible for neural plate differentiation.^2^ Facial malformations of varying degrees, deficiency in hormone production (mainly antidiuretic hormone), dysfunctions and problems in maintaining homeostasis (regulating temperature, controlling heart and respiratory rate) can also occur.

HPE occurs in four different degrees of intensity, as shown in Table 1.^3^ HPE is found in approximately 1:16,000 of live births, and the causes are still poorly understood. Although the fundamental cause is unknown in 70% of cases, currently, in addition to trisomy 13, different genes responsible for HPE are known through microdeletions or microduplications, such as: sonic hedgehog (SHH), zinc-finger protein (ZIC2), SIX homeobox (3SIX3), induced factor homeobox (TGIF), induced factor homeobox 1 (TGFß), patched gene family (PTCH) (receptor for the sonic hedgehog gene), GLI family zinc-finger 2 (GLI2) and teratocarcinoma-derived growth factor 1 (TDGF1). In unknown cases, it is assumed that the genetic defect is multicausal, involving multiple genetic and environmental factors, such as maternal diabetes and fetal exposure to ethanol. In this sense, HPE behaves as an autosomal dominant syndrome with variable penetrance and variability.^4^

**Table 1.** Degrees of intensity for holoprosencephaly.^3^

Alobar	More severe form, in which there is no division between the cerebral hemispheres. There is a single, small ventricle and complete absence of the corpus callosum and olfactory bulb.
Semilobar	There is partial separation of the cerebral hemispheres, absence of the corpus callosum, hypoplasia or absence of the olfactory bulb.
Lobar	There is a distinction between cerebral lobes and division of the cerebral hemispheres. Corpus callosum may be absent, hypoplastic, or normal.
Medium Inter-hemispheric Fusion	It presents failure in the division between the frontal and parietal lobes, absence of corpus callosum and heterotopic gray matter.

In this case report, the relationship between the two described pathologies is evident. In fact, 24 to 45% of Patau syndrome cases are associated with HPE.^5^ Studies relate group D trisomy (genes contained between chromosomes 13 and 15), that is synonym for Patau syndrome, as the main cause of the emergence of HPE. Group D mosaicism, trisomy 10, Klinefelter syndrome and deletion of chromosomes 13 or 18 are also important causes.^6^

The aim of the present study is to analyze radiological (gestational and perinatal) and neonatal findings of a patient with Patau syndrome and semilobar HPE and to describe the association of both pathologies.

## CASE REPORT

**Figure 1.**
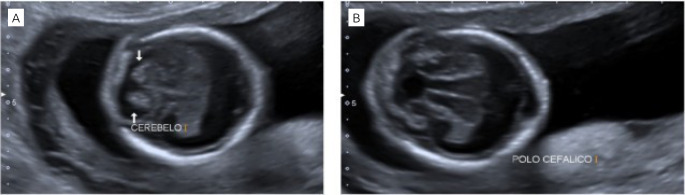
Obstetric ultrasound showing neurological alterations in Patau syndrome: (1A). Presence of a single ventricle and fusion of the thalamus; (1B). Cerebellar vermis agenesis.

**Figure 2.**
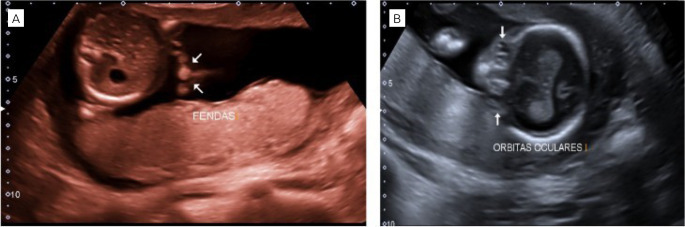
Obstetric ultrasound in Patau syndrome showing: (2A). Complex upper lip cleft; (2B). Hypotelorism with calcification in the eyeball;

**Figure 3.**
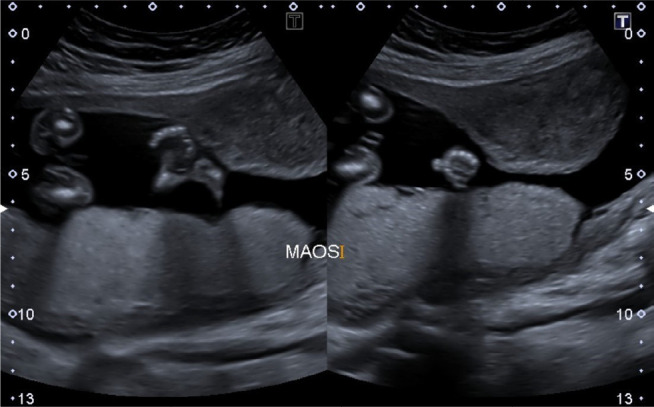
Obstetric ultrasound demonstrating bilateral polydactyly.

**Figure 4.**
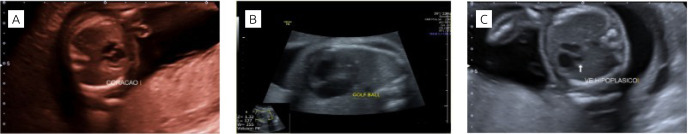
Obstetric ultrasound showing cardiac alterations in Patau syndrome: (4A). Calcification in the mitral valve chordae (golf ball); (4B). Left ventricular hypoplasia; (4C). Asymmetry of cardiac chambers in 4-chamber section.

This case report is about a female infant, born at term, from a woman aged over 40 years, in her third pregnancy, with no complications in previous pregnancies. In prenatal care, routine obstetric ultrasound was performed at 18 weeks and 3 days, which made evident findings of semilobar HPE with thalamic fusion and single first ventricle (Figure 1A), cerebellar vermis hypoplasia (Figure 1B), nasal hypoplasia, low implantation of the ears, bilateral incisive transforaminal cleft lip and palate^7^ (Figure 2A), hypotelorism with increased lens density (Figure 2B), bilateral polydactyly (Figure 3) of hands and feet, presence of golf-ball sign (calcification of the mitral valve chordae) (Figure 4A), and hypoplasia of left heart chambers, especially ventricular (Figure 4B). Regarding the other systems, it was confirmed: no closure defects in the vertebral column; intact abdominal wall, topical and normal-appearing stomach; usual-appearing liver and intestinal loops with standard echogenicity.

In addition, the report indicated a single fetus in a longitudinal position, cephalic presentation, with the anterior back showing active movement and present heartbeats, with a frequency of 153 bpm. Placenta was in Grannum's grade 0, thickness of 22 mm and amniotic fluid index within limits (AFI +11.3; largest pocket 33.3 mm). Therefore, the hypothesis of Patau syndrome was suggested for the fetus in question. A morphological ultrasound at 22 weeks showed the presence of a single umbilical artery.

The diagnosis was concluded at 21 weeks of gestation, after performing a GTG-banding karyotype in amniotic fluid: complete trisomy of chromosome 13 and pericentric inversion (considered normal) of chromosome 9 (47,XX,inv(9)(p12q13),+13).

The last ultrasound examination was performed at the 33rd week of pregnancy, in which it presented changes that further preserved the prognosis of the fetus. On examination, the fetus was in a longitudinal position, cephalad, with active movement and present heartbeats (frequency of 156 bpm). The placenta was in Grannum's grade I, located on the posterior body wall, thickness of 31.0 mm, and amniotic fluid index within the limits (AFI 16.1; largest pocket 62.3 mm).

In addition, it was demonstrated low weight (6th percentile) and decreased head circumference (3rd percentile) for gestational age, severe facial malformations and heart disease, with increased dimensions of the right heart chambers (Figure 4C), myocardial hypertrophy and mild pericardial effusion, in addition to hepatic vein ectasia, suggestive of systemic congestion.

Cesarean delivery took place at 37 weeks and 5 days, after the mother went into labor. There was meconium in the amniotic fluid.

The newborn, female, born at term with 46 cm and 3,066g, presented also deformation in the external auditory pavilion, in addition to the ones described by the USG. The family chose to prevent the patient from being admitted to the ICU, allowing only nasoenteral tube feeding in an incubator. The patient did not require oxygen therapy.

Due to cardiac complications, caused by Patau syndrome, the patient suffered cardiorespiratory arrest and died 12 hours after birth. Death certificate showed Patau syndrome as the cause of death.

## DISCUSSION

One of the main risk factors for the emergence of Patau syndrome is age over 35 years.^8^ In the case described, the patient is over 40 years old, significantly increasing the risk of chromosomal non-disjunction and consequently of Patau syndrome and other more common chromosomal syndromes such as Down and Edwards.^9^

Patau syndrome can be caused by mosaicism, complete trisomy or even by translocation of chromosome 13. As a consequence, there are several anomalies that often culminate in incompatibility with life. Among them are mainly neurological, cardiac, and urogenital alterations, which contributes to a very reserved prognosis.^8^

In prenatal care, the diagnostic hypothesis of Patau syndrome is raised mainly by the presence of abnormalities in fetal formation on ultrasound examination, being confirmed with karyotype examination. One should think about Patau syndrome, fetuses with growth restriction, defects in neural tube closure, cardiac and renal malformations, HPE, among others, in addition to late maternal age.

HPE derives from the intense malformation of the forebrain in fetal development, causing non-separation of the cerebral hemispheres in the frontal lobe. In the case described, incomplete forebrain cleavage manifested itself in what is classified as semilobar, that is, despite a tendency of hemispheric separation, this did not occur, also resulting in the fusion of the thalamus and the first right and left ventricles of the brain. It is worth mentioning that individuals with such deformity tend to present cognitive delay, so, even if the individual exceeds the restricted life expectations related to Patau syndrome there would be intense intellectual retardation.^10^

The fetus described in the case evolved with affected development, in addition to cardiac changes that led to severe complications compromising its prognosis. Regarding the patient's birth weight, it is noteworthy that, since the calculation for estimated fetal weight uses the head circumference as a variable, it was probably underestimated by this diagnostic method, explaining the 6th percentile observation, even though the weight was normal.

Despite medical advances in early diagnosis and treatment, the prognosis for this syndrome remains very unfavorable, and in most cases, spontaneous abortion occurs. Those who are born have a short life expectancy and the 10% of patients who are able to reach the first year of life have numerous adverse health conditions associated^11^. Considering this information, the ethical aspect of the case gains importance, that is, due to the high mortality of the described pathology, it is important that the physician responsible for the case gives full autonomy so that the family can make active decisions in the management and follow-up. In this case, the Do-Not-Resuscitate order was issued by the parents, who also requested that the patient would not be admitted to the ICU. In addition, multidisciplinary support, specifically family psychological support, is also significant.

It is essential that, in this situation, the physician in charge knows how to deal with the continuation of the case, considering the high rate of fetal mortality and the multiple malformations related to trisomy 13.^12^

In view of the discussion, it is concluded that Patau syndrome is one of the main causes of non-development of the forebrain with consequent fusion of the hemispheres in the frontal lobe. However, the mechanism between these pathologies is not clearly elucidated, and a significant part of the cases are associated with HPE.

The present report elucidates a case of a neonate diagnosed with Patau syndrome associated with semilobar HPE during prenatal care. In this way, the uniqueness of the work lies in the quest to understand the relationship between such diseases and to discuss the most sensible actions to be taken to promote better expectations for this class of patients.
